# A Description for Rock Joint Roughness Based on Terrestrial Laser Scanner and Image Analysis

**DOI:** 10.1038/srep16999

**Published:** 2015-11-20

**Authors:** Yunfeng Ge, Huiming Tang, M. A. M Ez Eldin, Pengyu Chen, Liangqing Wang, Jinge Wang

**Affiliations:** 1 Faculty of Engineering, China University of Geosciences, Wuhan, Hubei 430074, China; 2 College of Petroleum Geology and Minerals, University of Bahri, Khartoum, 1660/11111, Sudan; 3 Three Gorges Research Center for Geo-hazard, Ministry of Education, China University of Geosciences, Wuhan 430074, China

## Abstract

Shear behavior of rock mass greatly depends upon the rock joint roughness which is generally characterized by anisotropy, scale effect and interval effect. A new index enabling to capture all the three features, namely brightness area percentage (*BAP*), is presented to express the roughness based on synthetic illumination of a digital terrain model derived from terrestrial laser scanner (TLS). Since only tiny planes facing opposite to shear direction make contribution to resistance during shear failure, therefore these planes are recognized through the image processing technique by taking advantage of the fact that they appear brighter than other ones under the same light source. Comparison with existing roughness indexes and two case studies were illustrated to test the performance of *BAP* description. The results reveal that the rock joint roughness estimated by the presented description has a good match with existing roughness methods and displays a wider applicability.

Joint roughness is one of the most important parameters for understanding mechanical behavior and permeability characteristics of rock mass. Various quantificational indexes have been proposed for describing the degree of joint roughness, they can be classified into three main categories: joint roughness coefficient (JRC)^1^,^2^, statistical parameters^3^,^4^,^5^,^6^,^7^, and fractal dimension^8^,^9^,^10^,^11^,^12^,^13^. Although these indexes has been widely used, yet they have some limitations, for example, the choice of JRC value is highly subjective and unreliable through visual comparison with standard profiles, and majority of statistical parameters are calculated based on 2D profile to represent roughness in the 3D level that leads to biased and incomplete representation. Generally, the fractal dimension D is able to capture the self-similarity of natural rock joints, but it cannot estimate anisotropy of rock joints, because the effect of direction is not considered in the calculation of D value. Since Barton firstly introduced JRC to describing rock joint roughness in 1973^1^, researchers have made great progress over the past four decades in conceptualizing the terms of roughness estimation. It has been widely recognized that rock joint roughness is always characterized by anisotropy, scale effect, and interval effect. The sections below present a summary of state-of-art on these three aspects on which this paper mainly concentrates.

The anisotropy of rock joint roughness has been investigated by many researchers^14^,^15^,^16^,^17^,^18^,^19^,^20^,^21^,^22^,^23^,^24^,^25^,^26^, from their natural or artificial rock joint samples in 2D or 3D level. Their findings showed that roughness of a rock joint varies with direction where the profile or surface geometry data was extracted. The direction of roughness estimation has to be chosen along movement or water seepage direction, because of significant direction dependence of roughness.

To estimate the scale dependence of rock joint roughness, the rock joint roughness has be investigated with different sampling length or window based on various indexes such as JRC, statistical parameters (the root mean square of the slope of the profile *Z*_*2*_, the mean inclination angle of the profile *θ*_*p*_, etc.), and fractal dimension D^2^,^27^,^28^,^29^,^30^,^31^,^32^,^33^. Their findings reveal that roughness indexes are scale-dependent and their values decrease or increase with the increasing of sampling size, but reach to a constant level when sampling size is larger than a threshold. Such increase trend always found at rock joints with narrow range of sampling size or less than 3.0 m^31^ is ordinarily termed as positive scale effect, On the contrary, decrease trend is named as negative scale effect for larger or wider sampling size of rock joints. As a result of these contradictory statements, the scale dependence of rock joint roughness remains a subject under continuous debate.

Sampling interval exhibits a close relationship to measurement resolution, the smaller the interval, the higher the resolution. In order to examine the impact of sampling interval on rock joint roughness, it is imperative to digitize the a certain joint profile or surface at different measurement resolution. In the recent years, only a fewer authors have paid attention to this study field^34^,^35^,^36^. In addition, the range and number of sampling intervals are always not large enough. Basically, they hold at this point that capturing of more geometrical information and decrease of sampling interval, the rock joint is expected to have higher roughness. However, it is a big consumption of time and cost to digitize the rock joints using measurement with smaller sampling interval. The choice of a suitable sampling interval is the key to improve the efficiency of practice application.

From the reviews mentioned previously, we conclude that the sampling direction, scale, and resolution can greatly affect the estimation of rock joint roughness, due to these reasons and their own inherent defects, it is difficult to make an accurate and comprehensive evaluation on rock joint roughness using the existing three kinds of methods. Therefore, to make up their imperfections, it is necessary to present a new index enabling to provide a quantitation of joint roughness according to 3D geometry data. In addition, this index is expected to have the capability of capturing all three aforementioned features. In this paper, an index, brightness area percentage (*BAP*), is proposed to quantify rock joint roughness based on the terrestrial laser scanner and image analysis.

## Methodology

Under the irradiation of light, the rock joint surface shows different brightness levels due to the asperity. From micro-scale points of view, the joint surface consists of numerous tiny planes, the degree of brightness of these tiny planes can be affected directly by their orientation (dip angle and dip direction) and elevation. The steep portions of the joint facing the light source display more luminous than that located at the back to the light source, and at the same time the brighter planes play an important role in the resistance during shearing failure, so variation and difference in brightness level can reflect the rock joint surface roughness very well. Consequently, in this study the estimation of roughness is focused on those kinds of tiny planes in rock joint surface. The term *BAP* is defined as the index to evaluate the rock joint surface roughness and can be carried out through four steps: (1) data collection; (2) Data processing and model building; (3) light source simulation; and (4) calculation of *BAP*. A detailed description of the four steps is addressed in the following subsections.

### Data Collection

**Figure 1:**
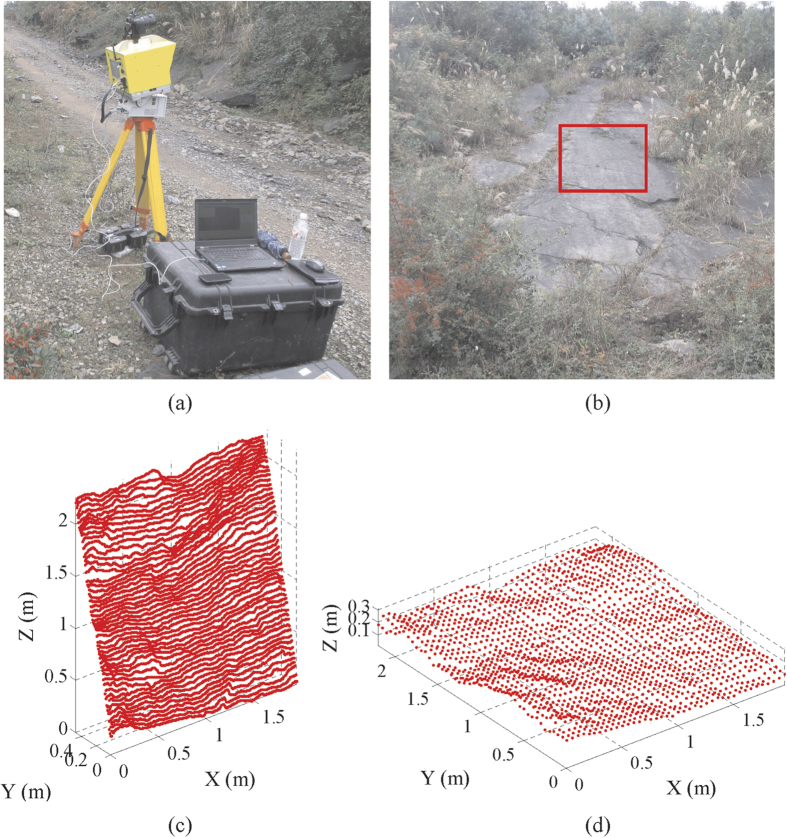
*In situ* natural rock joint surface selected for 3D laser scanning tests (**a**) Field configuration of 3D laser scanner. (**b**) Removing the vegetation to reduce scanning interference. Difference between the (**c**) non-stationary surface and (**d**) stationary surface after removal of the global trend for the *in-situ* rock joint sample LS09.

In this study, the ILRIS-3_6_D terrestrial laser scanner was utilized to digitize the rock joint surface, marked as LS09, collected from Jiweishan rockslide area in Wulong County, Chongqing City, China (Fig. 1(a)). Different sampling windows and sampling intervals were performed to obtain the point cloud for the same rock joint. The red rectangle indicates the scanning zone where the roughness data was collected (Fig. 1(b)). To meet the requirement of impact analysis of different sampling parameters, a series of data collections in different sampling windows and resolution were carried out. To remove errors due to any movement of the scanner platform during this investigation, special consideration has been given to acquire one scan at the maximum point density and coverage required, and then the data are classified into different intervals and scales. The sampling windows have different sizes (length × height) ranging from 0.1 × 0.1 m to 1.90 × 1.90 m, and sampling intervals vary from 0.01 m to 1 m. The joint surface is discretized into a large number of points called Point Cloud, and each point is represented by space geometry of XYZ coordinates.

### Data Processing and Model Building

Generally, the rock joint surface roughness consists of two components: high-frequency small scale of roughness and low-frequency large scale of waviness. The roughness which describes the finer irregularities in the surface is considered as the stationary component, and waviness (non-stationary component) upon which roughness is superimposed indicates the global trend of the rock joint surface^37^,^38^. The global trend is represented by the fitting plane through the cloud of points on rock joint surface. The fitting plane can be found based on the least squares regression analysis. In practice, both components determine the strength, deformability and hydraulic behaviors of jointed rock mass. Two components of roughness always need to be estimated separately. The inclination angle *I* of the fitting plane is proposed to capture the non-stationary component at the simplest level^37^. Existing methods dealing with rock joint roughness are mostly concerned with the stationary component, Barton’s 10 typical profiles are stationary^1^. Also, research results suggest that the importance of removal of non-stationary component to obtain accurate estimates for the fractal parameters^39^. Furthermore, the global trend of joint roughness in the laboratory is different from that in field due to the sample disturbance. Therefore, the method proposed in this paper also estimates the rock joint roughness based on stationary component, to obtain reliable estimates for roughness, it is an important step to remove the global trend so as to obtain stationary roughness, as shown in Fig. 1(c,d), detailed information on size effect and removal of global trend can be found as Supplementary Fig. S1 and S2 online.

Measurement noise inherent in the point cloud will be introduced during scanning; the noise may arise from external and internal factors. External factors mainly relate to the obstruction such as thin vegetation (Fig. 1(b), Supplementary Fig. S3(a,b)), by specifying a threshold, the points whose distance beyond threshold can be edited out automatically, alternatively, the points of thin vegetation may be deleted manually. On the other hand, the internal factors mainly originates from the imprecision of the scanning mechanism and the physical and geometric properties of the rock joint surface itself. Wavelet transformation was employed to reduce this type of noise in the raw point cloud. Thus, a thorough treatment of noise is essential to acquire realistic characterization of rock joint roughness. In the case of most points with size of 1.90 × 1.90 m and an interval of 0.01 m, there exist approximately 37000 points in the raw data totally, nearly noisy 900 points were removed based on above-mentioned two de-noising methods. Subsequently, the rock joint surface was reconstructed from the de-nosied point cloud data based on a triangulation algorithm, and the joint surface was discretized into contiguous triangles and keep a good integrity (see Supplementary Fig. S4 online).

### Light Source Simulation

**Figure 2:**
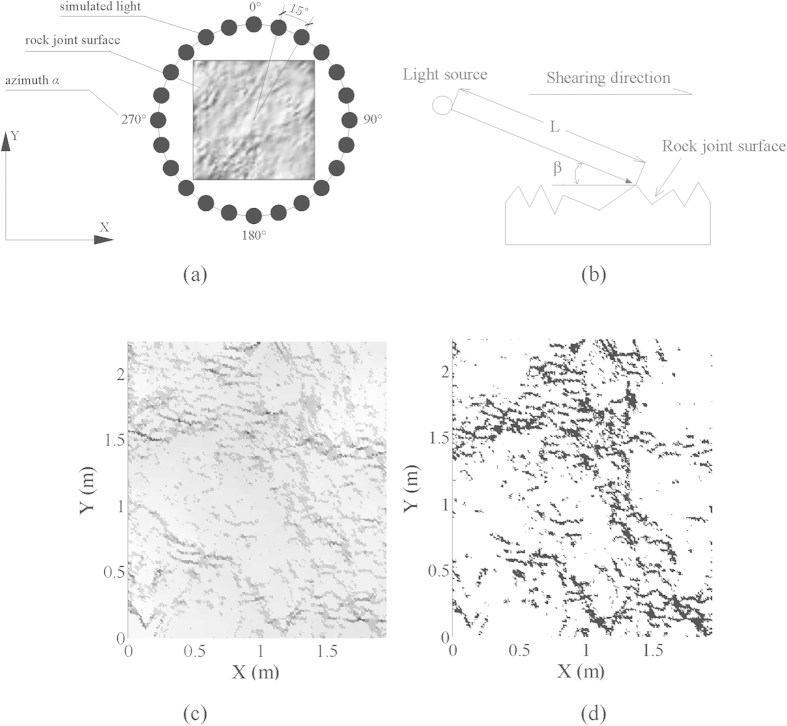
The diagram shows the required parameters in light source simulation: (**a**) Azimuth angle *α* and (**b**) Incidence angle *β*. The (**c**) Gray scale image and (**d**) Bilevel image is generated based on image techniques.

For the purpose of generating the brightness and shadow on the joint surface, the physical light source is needed to illuminate the joint surface. Ideally, the physical light source should be small, bright, and far enough to emit parallel rays for precise measurement^5^. Nevertheless, the physical light experiment is time-consuming and in inconvenient. So instead of physical light source, a virtual light source was simulated to generate parallel light and irradiate the rock joint surface. In terms of light source simulation, two parameters, i.e. azimuth angle *α* and incidence angle *β* which is the angle between the light source and the horizontal plane, were specified according to the study interests. The anisotropy of rock joint roughness was analyzed via changing the value of azimuth angle *α* in step of 15° from 0° to 360°. The incidence angle *β* was assigned in the range of 35° to 70°, in which the calculated result can vary from sample to sample in a better way^40^. Figure 2(a,b) defines the former two parameters. Note that, although both parameters *L* and *S* are related to light distance and strength, they were assumed as a constant value for each study case because of slight range of these two parameters. Also, special attention should be paid to the setting that direction of light should be arranged identical with shear direction (see Fig. 2(b)).

### Calculation of *BAP*

Only the tiny steep planes are facing shear direction can keep in contact during shearing. If the light irradiates at the same direction with shearing, the tiny planes which are opposite to shear direction will be identified as bright areas. In the whole bright areas, the tiny planes are further distinguished by their brightness level, which represents different dip angle. The higher the brightness level, the steeper the dip angle of the tiny plane on the rock joint surface. The distribution of luminance on the joint surface is generated and saved as the format of digital gray scale image. Each pixel includes only one gray scale in the image. The gray scale ranges from 0 to 255 and indicates the brightness degree of the tiny planes. The gray level of 0 means no brightness and color is black, accordingly, the total white color has a gray level of 255 (Fig. 2(c)). 160 was chosen as a threshold of gray level *TL* which depends much on the normal load applied on the rock joint, larger normal load indicates more tiny planes keep contacted, so the smaller threshold will be specified, it is reported that the threshold that is required decreases with the increasing of normal load (Supplementary Fig. S5 and S6 online)^41^. The tiny planes with gray level more than 160 have steep enough dip angles and keep in contact. According to the threshold gray level, the gray scale image is converted into bi-level image, in which white color represents the contact area and black color represents non-contact area (Fig. 2(d)).

*BAP* can be computed according to the following equation,


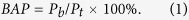


where, *P*_*b*_ is the amount of pixels of the white color area and *P*_*t*_ is the total number of pixels for the whole joint area in the bi-level image, respectively.

## Results and Discussion

### Comparison with existing roughness indexes

To verify the reliability of the proposed method of estimating the rock joint roughness, the anisotropy, scale effect and interval effect of joint roughness were investigated. There are some resemblances between *BAP* method and 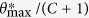 method that proposed by Grasselli (2002)^7^, where 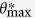 is the maximum apparent dip angle along the shear direction, and *C* is a roughness parameter. Both methods take tiny steep planes facing shear direction as investigated objects, but the ways to identify these planes are different. To examine the robustness of *BAP* method, Grasselli’s and fractal methods were employed to evaluate the roughness of same rock joint, and comparison is performed between them.

**Figure 3:**
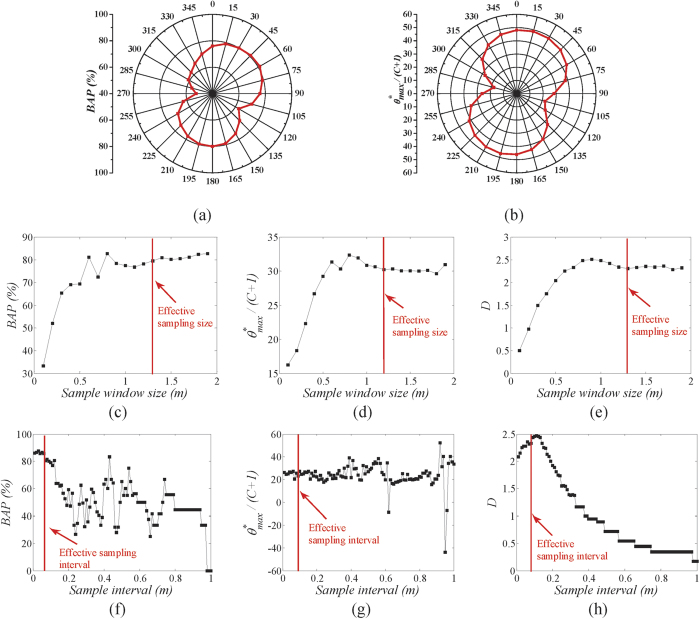
The variation of (**a**) *BAP* & (**b**) 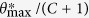 with investigated direction. The relationships between (**c**) *BAP*, (**d**) 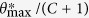, (**e**) *D* and sampling size, note that the X axis represents the length of side of sampling window. The relationships between (**f**) *BAP*, (**g**) 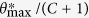, (**h**) *D* and sampling intervals.

The gray scale images of different directions were obtained for the anisotropy analysis. The distribution of brightness and darkness is varied gradually with the changing of azimuth angle α. The values of *BAP* and 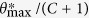 in different directions range from 0° to 360° were calculated, and plotted on the radar chart as shown in Fig. 3(a,b). It is obvious that the two results based on *BAP* and 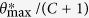 have experienced analogical variation tendencies to a certain degree. The rock joint roughness around 0° and 180° directions are larger than that in 90° and 270°.

For the scale effect analysis, 19 concentric square-shaped sampling windows were extracted from the rock joint sample LS09. The minimum window size is 0.1 × 0.1 m and maximum is 1.9 × 1.9 m with an equivalent in side length increment of 0.1 m. The scale effect of joint roughness for different sampling size was estimated by calculating *BAP*, 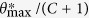 and D based on the above mentioned methods. Figure 3(c–e) indicate the variation of *BAP*, 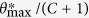 and D with the increasing of sampling window size, respectively. Their results have a good matching and are classified into the category of positive scale effect, all three roughness indexes increase with sampling window size, and effective sampling sizes for the three cases are similar to each other, approximately 1.2 × 1.2 m.

For interval effect analysis, 100 cases of rock joint roughness data from different intervals ranging from 0.01 m to 1.0 m were collected for the rock joint sample LS09. Analogous to the previous research results on this topic^33^,^36^, the joint roughness decreases with the sparsity of sampling interval. The values of *BAP*, 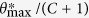, and *D* were obtained from different resolution and to evaluate the interval effect of joint roughness. Figure 3(f) illustrates that the *BAP* increases with the decreasing of sampling intervals, when the interval is less than 0.08 m, it remains almost stable and fluctuate around 85%. In contrast, the fractal dimension *D* also has similar changing trends, but the effective sampling interval is 0.09 m. Although the curve of 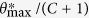 shows the changes in a different way, the amplitude of variation in the range intervals of 0.01–0.11 m is less than that in the range of 0.11–1.00 m. 0.11 m can be considered as the effective sampling interval (Fig. 3(g,h)). Therefore, the smaller the sampling interval, the more reliable the results. Meanwhile, the effective sampling interval can be chosen to save time during 3D scanning testing.

### Application of *BAP* on two cases

**Figure 4:**
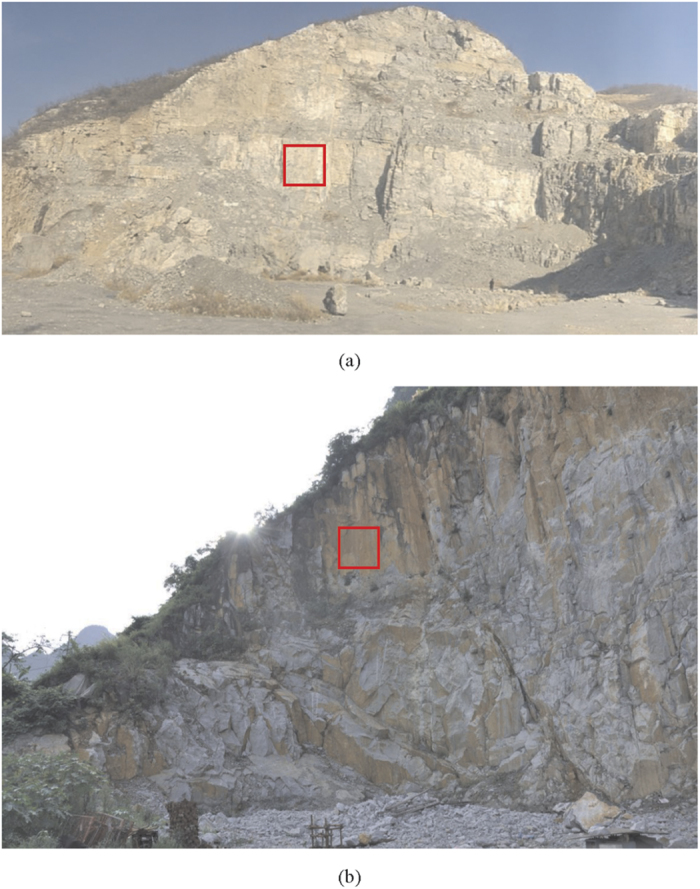
Rock exposure for terrestrial laser scanning. (**a**) Case A is an abandoned mine located in Henan, China. (**b**) Case B is a mining rock slope situated in Guangxi, China.

Two case studies were performed at two mining rock slopes for measuring the point clouds of large joint surface. To validate the applicability of the presented method (see Fig. 4(a) for Case A, and Fig. 4(b) for Case B), fractal dimension D and *BAP* were used to estimate rock joint roughness in these two cases. Table 1 shows the details of Case A and Case B with regard to the location, lithology, and discontinuity type. The same sampling size and interval were specified for the two cases to remove the effect from measurement scale and resolution.

**Figure 5:**
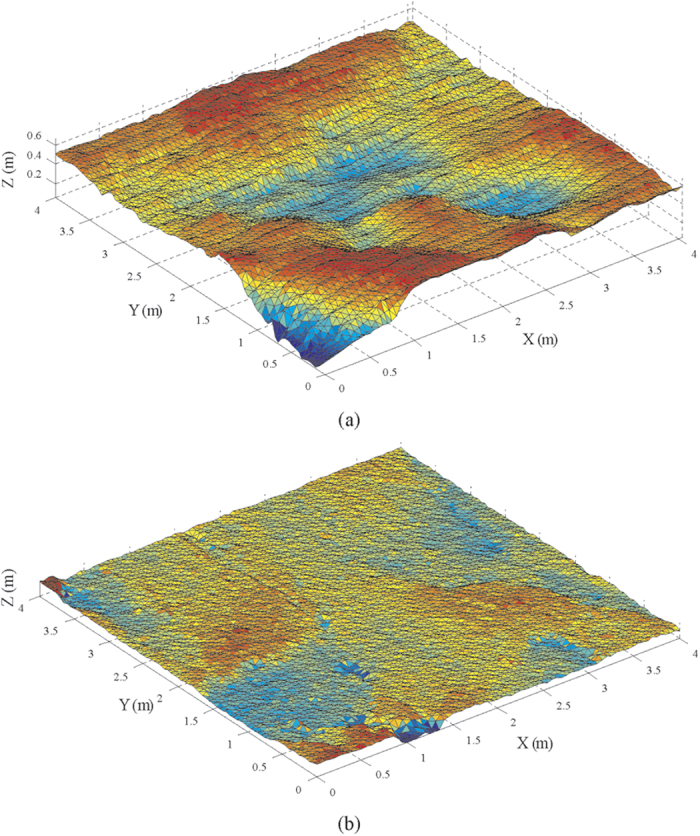
Reconstructed digital model of rock joint surface for (**a**) Case A and (**b**) Case B based on triangularization.

Two square areas with size of 4 × 4 m were selected as shown by the red windows in Fig. 4 for estimation of rock joint roughness using indexes of D and *BAP*. Through the data processing, de-noising and removing global trend, Fig. 5(a,b) present the two digital models of rock joint surface in Case A & B that were used for analysis. Based on the visual perception, the roughness level of joint surface in Fig. 5(a) (Case A) seems to be higher compared to that in Fig. 5(b) (Case B).

Values of *BAP* were calculated for both cases with same azimuth angle *α* (90°), incidence angle *β* (55°), and threshold of gray level *TL* (160). In addition, fractal dimension *D* of the two cases is also computed to analyze the joint roughness. Table 2 gives the comparison of analytical results based on *BAP* and *D* methods. Apparently, the results matched with each other very well, and results of both methods indicate that Case A has a higher roughness level than Case B.

### Effect of sampling size on global trend

**Figure 6:**
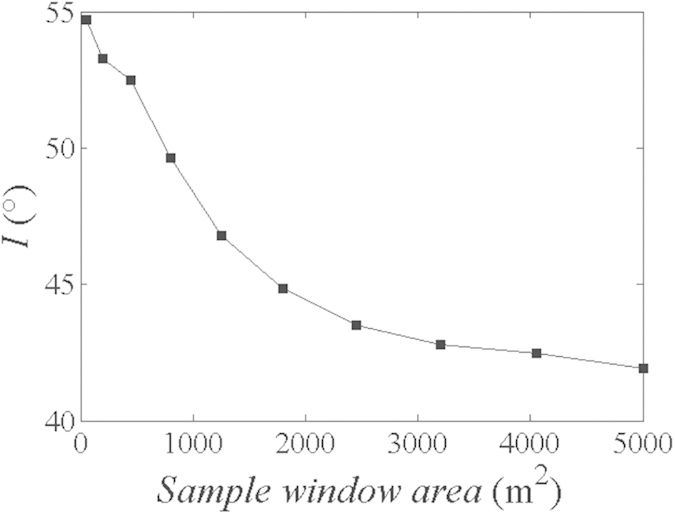
The relationship between the inclination angle *I* and sampling size.

Prior to this study, it has been reported that global trend is related to non-stationary component of roughness, and can be captured by average inclination angle *I*^37^. Roughness estimation using *BAP* should be conducted based on the point cloud after removal of global trend. Therefore, removal of global trend is regards as a key step to estimate roughness accurately. To investigate the scale dependence of the global trend, varying sampling windows from the large-scale rock outcrop in Case A were analyzed. The maximum size was specified as 50 × 100 m, and minimum one is 5 × 10 m. Subsequently, the fitting planes of point cloud of each window were captured based on the least square method, and *I* was calculated. Figure 6 shows the variation of *I* with sampling window area, and *I* has a gradual decrease trend with the expanding sampling window. Whilst, the results also suggest that the level of global trend in large size of rock joint tend to be gentler than that for smaller rock joint.

## Conclusions

Based on the observations and analyses presented in this paper, the major summaries and conclusions regarding the description of rock joint roughness are as follows:

3D geometrical data of joint surfaces are required to avoid distortion and loss of original information of roughness. the TLS was successfully employed *in situ* to measure a large scale joint surface in different sizes and intervals. TLS scanner is able to capture data in a short time and with high accuracy, which is essential for estimating roughness.

*BAP* presented in this study has been proved to be a reliable index of rock joint roughness. It has the capability to identify precisely these tiny planes from whole joint surface which keeps in contact and make more contribution during shearing. More importantly, the features of roughness in terms of anisotropy, scale effect and interval effect can be characterized through *BAP*. The evaluation is reasonable and it has good matches with other methods.

Roughness of natural rock joint surface significantly depends on the sampling parameters including direction, scale and resolution. Therefore, the direction where joint roughness was investigated must be the same for shear direction, effective sampling size and interval should be selected to eliminate scale and interval effect on roughness. For example, in this study, to capture real roughness of rock joint sample, sampling size and interval should be specified more than 1.3 × 1.3 m and less than 0.08 m based on *BAP* method, respectively. Note that the rock joint sample LS09 stated in this paper has a positive scale effect on roughness.

To investigate rock joint roughness accurately, the non-stationary component (global trend) is needed to be removed. Moreover, the global trend decreases with the increase of sampling size.

## Additional Information

**How to cite this article**: Ge, Y. *et al.* A Description for Rock Joint Roughness Based on Terrestrial Laser Scanner and Image Analysis. *Sci. Rep.* **5**, 16999; doi: 10.1038/srep16999 (2015).

## Supplementary Material

Supplementary Information

## Figures and Tables

**Table 1:** Geological settings and scanning parameters of Case A and Case B.

Case No.	Location	Lithology	Type	Scanning size	Scanning interval
				m × m)	(m)
Case A	Henan, China	limestone	joint	4 × 4	0.05
Case B	Guangxi, China	dolomite	joint	4 × 4	0.05

**Table 2:** Roughness estimation results for two cases based on *BAP* and *D* methods.

Case No.	*BAP* (%)	*D*
Case A	75.04	2.1557
Case B	68.88	2.1194
