# Supplementary material for: A Description for Rock Joint Roughness Based on Terrestrial Laser Scanner and Image Analysis
Source: Sci Rep. 2015 Nov 20;5:16999. doi: 10.1038/srep16999 (PMC4653810; doi:10.1038/srep16999)
Supplement: Supplementary Information [file srep16999-s1.pdf]

SUPPLEMENTARY INFORMATION

A Description for Rock Joint Roughness Based on Terrestrial Laser Scanner and Image Analysis

Yunfeng Ge[1]*, Huiming Tang[2]*, Ez Eldin, M. A. M[3], Pengyu Chen[4], Liangqing Wang[5], Jinge Wang[6]

1* Faculty of Engineering, China University of Geosciences, Wuhan, Hubei 430074, China, Email: cug_gyf@foxmail.com, Ph: +86 135 5444 2358, Fax: +86 027 6788 3507

2* Faculty of Engineering, China University of Geosciences, Wuhan, Hubei 430074, China, Email: tanghm@cug.edu.cn, Ph: +86 136 0715 3663, Fax: +86 027 6788 3044

3 College of Petroleum Geology and Minerals, University of Bahri, Khartoum, 1660/11111, Sudan, Email: mutasimadam@hotmail.com

4 Faculty of Engineering, China University of Geosciences, Wuhan, Hubei 430074, China, Email: 79012983@qq.com

5 Faculty of Engineering, China University of Geosciences, Wuhan, Hubei 430074, China, Email: wlq027@126.com

6 Three Gorges Research Center for Geo-hazard, Ministry of Education, China University of Geosciences, Wuhan 430074, China, Email: 250824225@qq.com

SUPPLEMENTARY FIGURES

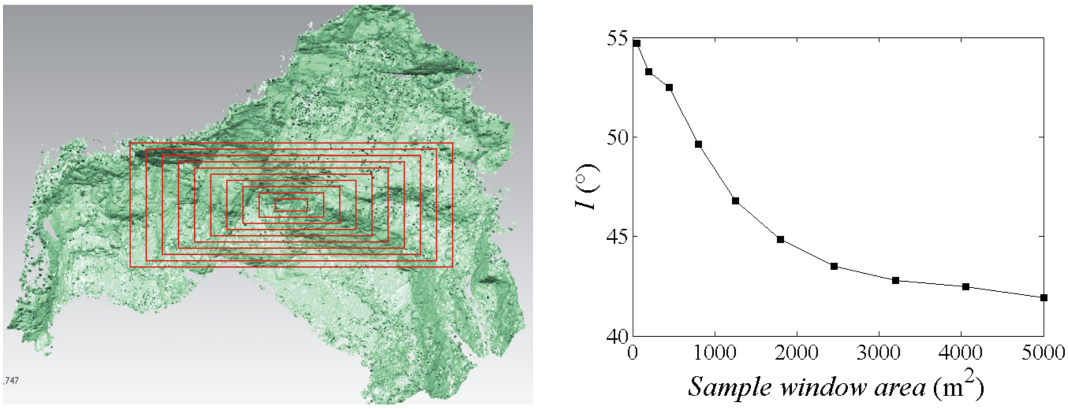

(a) (b)

Supplementary Fig. S1| The plot of (a) locations of different sampling windows for global trend analysis, and (b) relationship between the inclination angle I and sampling window size

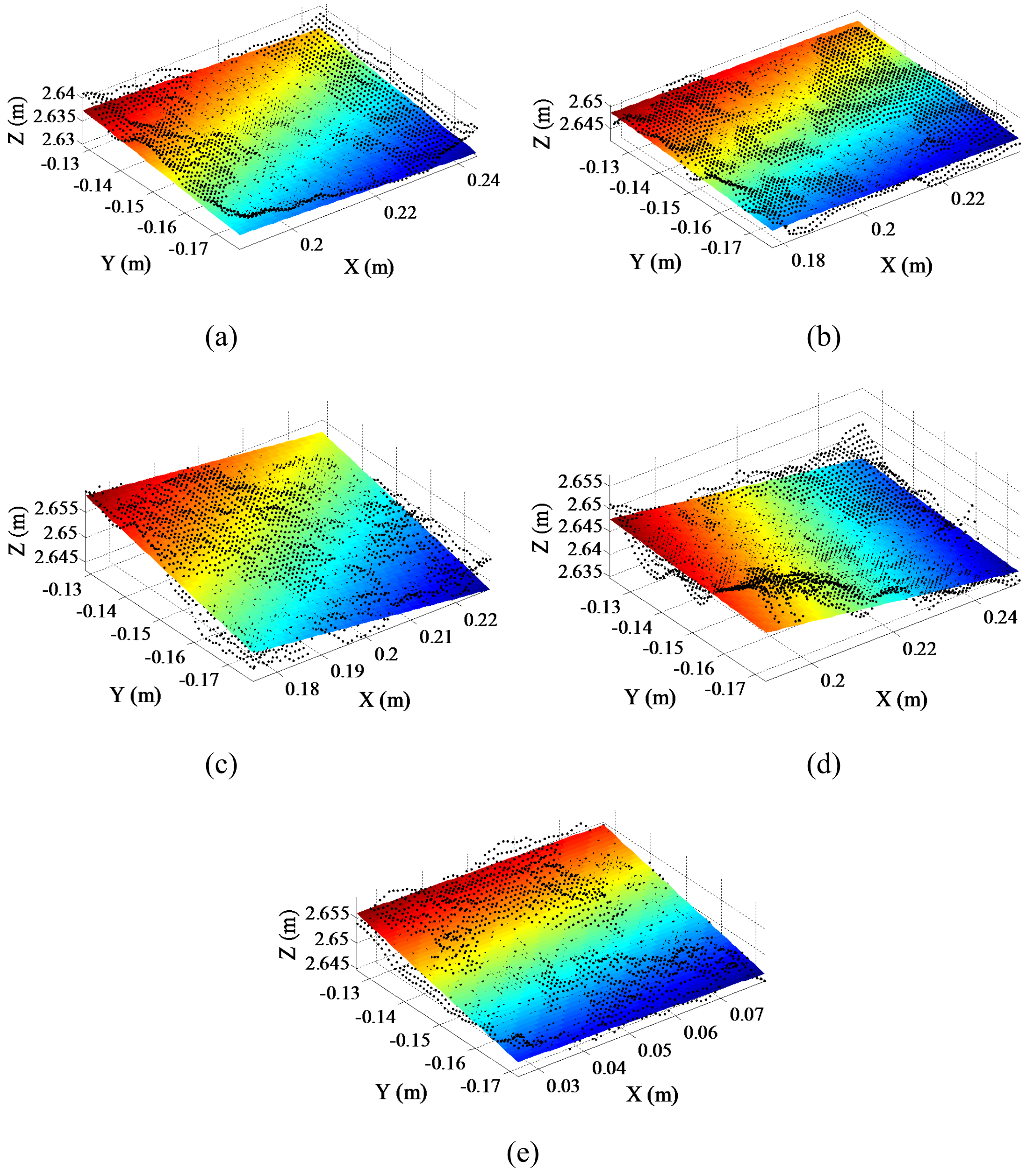

Supplementary Fig. S2| Pictorial view of point cloud and fitting planes for 5 rock joint samples

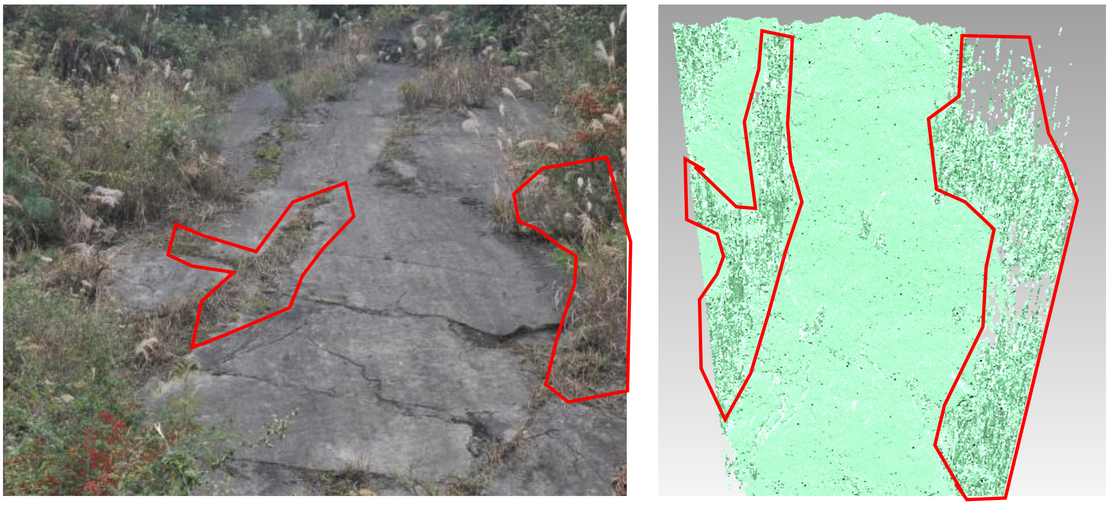

(a) (b)

Supplementary Fig. S3| The vegetation on the (a) in-situ large rock joint surface and corresponding (b) point cloud as shown the red polygon area

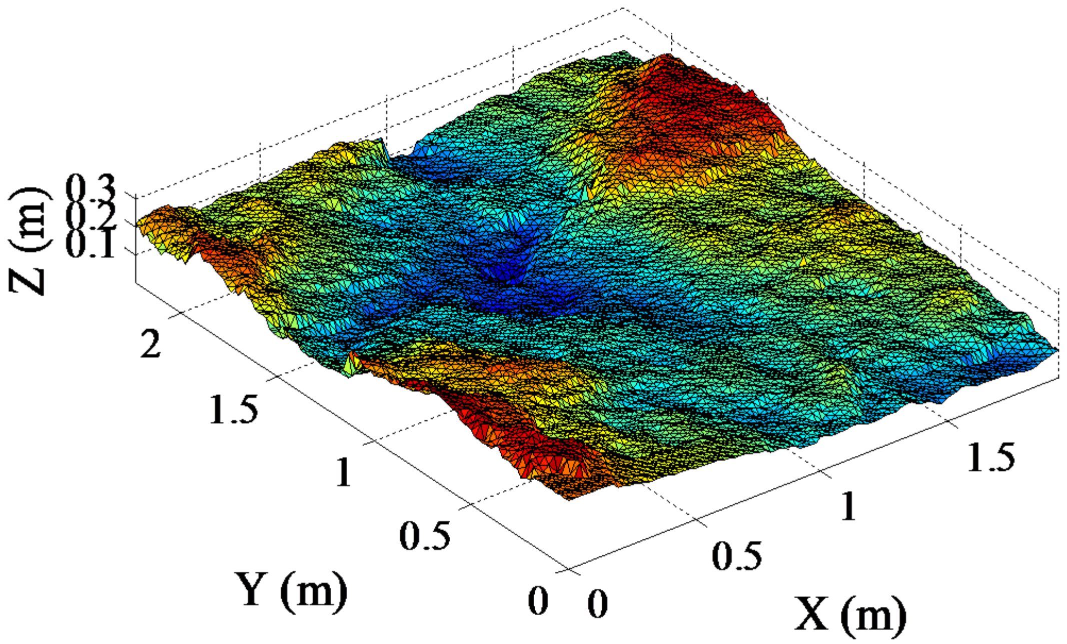

Supplementary Fig. S4| Reconstruction of joint surface was built from a triangular irregular network based the point data of removal noise

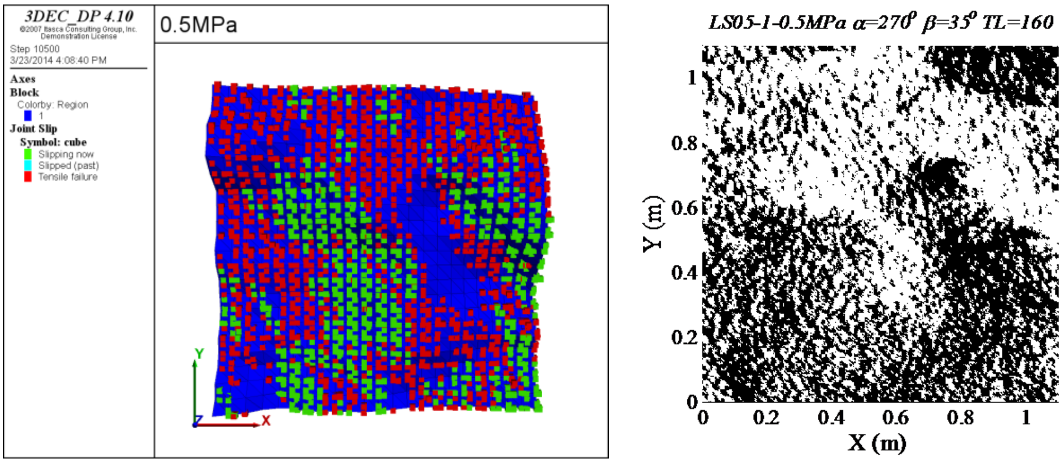

(a) normal loads = 0.5 MPa

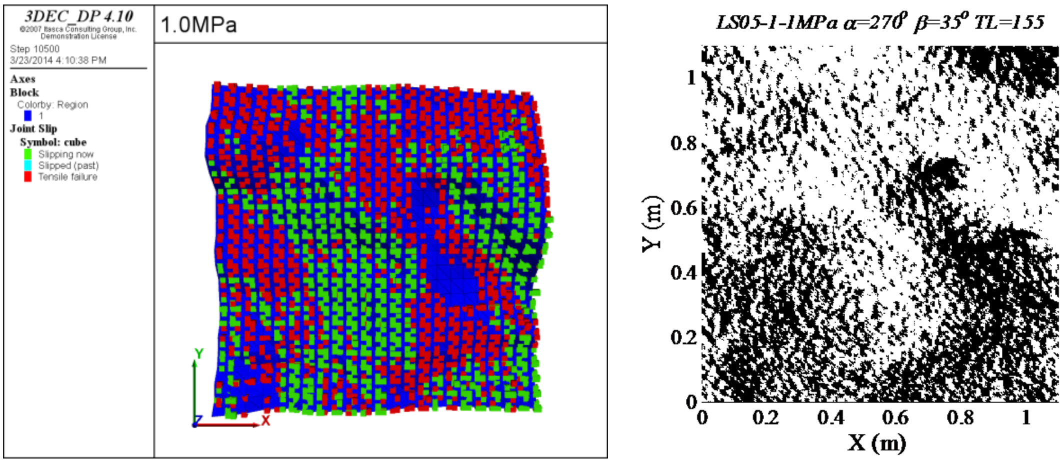

(b) normal loads = 1.0 MPa

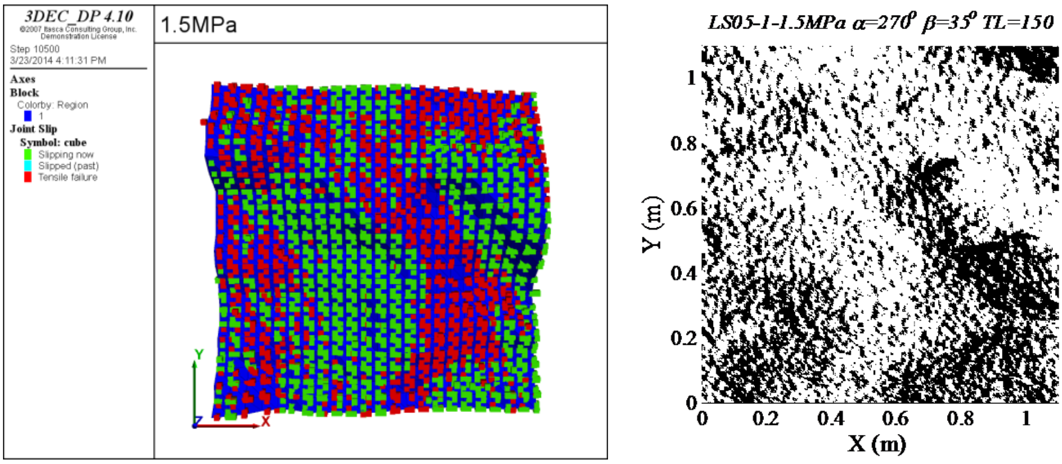

(c) normal loads = 1.5 MPa

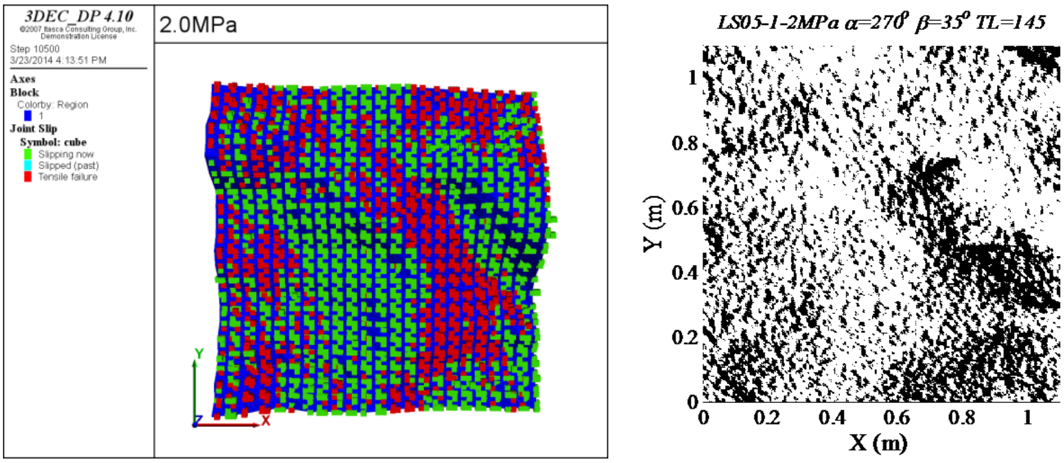

(d) normal loads = 2.0 MPa

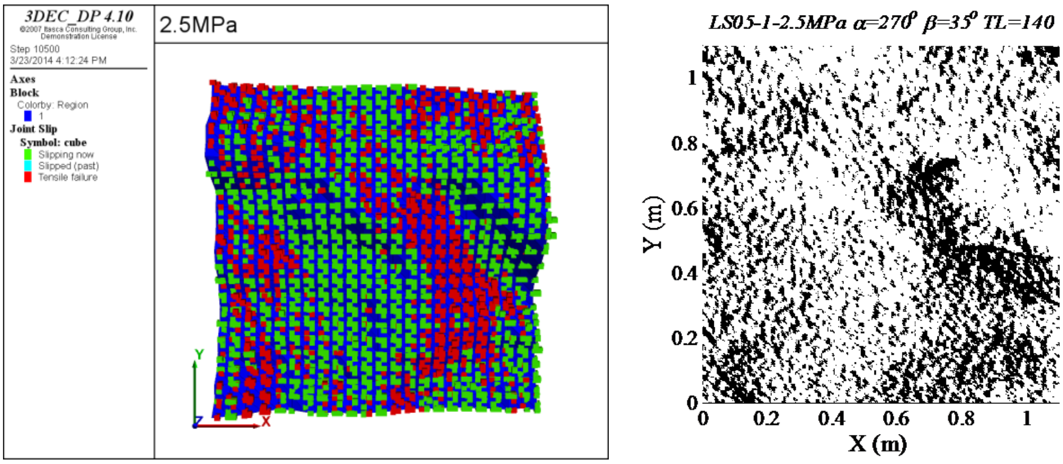

(e) normal loads = 2.5 MPa

Supplementary Fig. S5| Comparison of contact area estimation between simulation results and image analysis

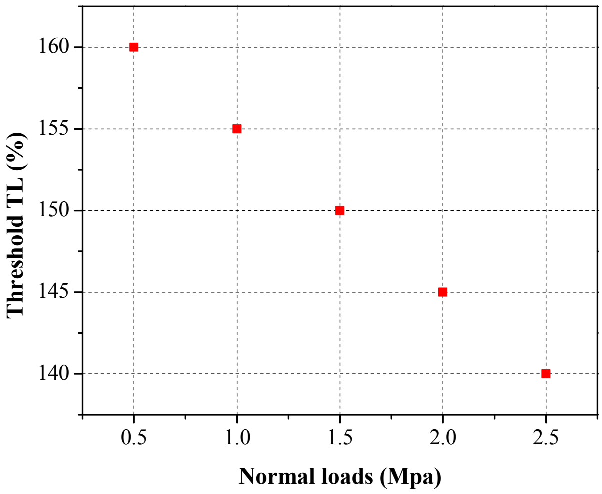

Supplementary Fig. S6| The relationship between threshold *TL* choosing for image analysis and normal loads applying to joint

SUPPLEMENTARY TEXTS

Prior to this study, it has been reported that global trend is related to non-stationary component of roughness, and can be captured by average inclination angle I[S1]. To examine the differences to the removal of the "global trend" step between large and small scanning area (scale effect of global trend), varying sampling windows from the large-scale rock outcrop in Case A were analyzed. The maximum size was specified as 50×100m, and minimum one is 5×10m (see Supplementary Fig. S1 (a)). Subsequently, the fitting planes of point cloud of each window were captured based on the least square method, and inclination angle I was calculated. Supplementary Fig. S1 (b) shows the variation of inclination angle I with sampling window size (in m^2), and inclination angle I has a gradual decrease trend with the expanding sampling window. Whilst, the results also suggest that the level of global trend in larger rock joints tend to be gentler than that for smaller ones.

The global trend can be defined through a two-dimensional plane. Generally, the rock joint surface roughness consists of two components: roughness and waviness. The roughness which describes the finer irregularities in the surface is considered as the stationary component, and waviness (non-stationary component) upon which roughness is superimposed indicates the global trend of the rock joint surface[S1,S2]. The global trend is represented by a fitting plane through the point cloud of joint surface. The fitting plane can be found based on the least squares regression analysis (see

Supplementary Fig. S2 (a)-(e)). As seen from Supplementary Fig. S2, the black points is the point cloud of rock surface, and plane represents the fitting plane based on the least square method.

Measurement noise inherent in the point cloud will be introduced during scanning, and the noise may arise from external and internal factors. External factors mainly relate to the obstruction such as thin vegetation (Supplementary Fig. S3 (a) & (b)), by specifying a threshold, the points whose distance beyond threshold can be edited out automatically, alternatively, the points of thin vegetation may be deleted manually. On the other hand, the internal factors are mainly originated from the imprecision of the scanning mechanism and the physical and geometric properties of the rock joint surface itself. Wavelet transformation was employed to reduce this type of noise in the raw point cloud. Thus, a thorough treatment of noise is essential to acquire realistic characterization of rock joint roughness.

For the scan with the maximum point density (interval = 0.01m) and coverage (1.90×1.90m), there are approximately 37000 points in the raw data totally, nearly 900 noisy points were removed based on de-noising methods. This scan area is located within the red box (see Figure. 1 (b) in the main article), and it can be observed that the places with few vegetation was chosen as interesting for avoiding external noise. Therefore, the point that need to be removed actually is very limited, resulting surface built from a triangular irregular network keep a good integrity (Supplementary Fig.

S4).

The selection of threshold much depend on the normal loads applying to the rock joint. To investigate the relationship between threshold *TL* and normal stress, estimation roughness and numerical simulation under different normal loads have been conducted by first author of this paper as shown in Supplementary Fig. S5 (a)-(e)[S3]. In the numerical simulation, the green dots indicate the portions of joint surface keep contact with other samples during shearing, and the areas non-contacted are represented by red dots. As mentioned in our manuscript, according to the threshold *TL*, the gray scale images are converted into bi-level images, in which white color represents the contact area and black color represents non-contact area. Through suitable selection of threshold *TL*, the areas covered by green dots can be equal to that occupied by white color. It can be clearly seen that threshold *TL* choosing for image analysis is in direct proportion to normal loads applying to rock joints (See Supplementary Fig. S6).

REFERENCES

S1. Kulatilake, P.H.S.W., Shou, G., Huang, T.H., & Morgan, R. M. New peak shear strength criteria for anisotropic rock joints. *International Journal of Rock Mechanics & Mining Sciences & Geomechanics Abstracts.* **32**, 673-697 (1995).

S2. Hong, E. S., Lee, I M., Cho, G C, Lee, S. W. New approach to quantifying rock joint roughness based on roughness mobilization characteristics. *Ksce Journal of Civil Engineering.* **18**, 984-991 (2014).

S3. Ge, Y. F., Kulatilake, P. H. S. W., Tang, H. M., & Xiong, C. R. Investigation of natural rock joint roughness. *Computers and Geotechnics.* **55**, 290-305 (2014).
